# Hospital admissions for asthma, diabetes and COPD: is there an association with practice nurse staffing? A cross sectional study using routinely collected data

**DOI:** 10.1186/1472-6963-10-276

**Published:** 2010-09-21

**Authors:** Peter Griffiths, Trevor Murrells, Dalia Dawoud, Simon Jones

**Affiliations:** 1 King's College London, National Nursing Research Unit, 57 Waterloo Road, London, UK; 2 Cairo University, Kasr El-Aini Street, Cairo, Egypt

## Abstract

**Background:**

Delivering good quality primary care for patients with chronic conditions has the potential to reduce non-elective hospital admissions. Practice nurse staffing levels in England have been linked to attainment of general practice performance targets for some chronic conditions. The aim of this study was to examine whether practice nurse staffing level is similarly associated with non-elective hospital admissions in three clinical areas: asthma, Chronic Obstructive Pulmonary Disease (COPD) and diabetes.

**Methods:**

This observational study used cross sectional analysis of routinely collected data. Hospital admissions data for the period 2005-2006 (for asthma, COPD and diabetes) were linked with a database of practice characteristics, nurse staffing data and data on population characteristics for the same period. Statistical modelling explored the relationship between non-elective hospital admission rates for the three conditions and the list size per full time equivalent (FTE) practice nurse.

**Results:**

Higher practice nurse staffing levels were significantly associated with lower rates of admission for asthma (p < 0.001) and COPD (p < 0.001). A similar association was seen for patients with two or more admissions (p < 0.05 for asthma and p < 0.001 for COPD). For diabetes, higher practice nurse staffing level was significantly associated with higher admission rates (p < 0.05), but this association was not significant in case of patients with two or more admissions. Across all models, increasing deprivation was associated with higher admission rates for all conditions.

**Conclusions:**

The inconsistent relationship between nurse staffing and patient outcomes across the different conditions and the fact that for diabetes the relationship between staffing and outcomes was in a different direction from the association between staffing and care quality, highlights the need to avoid making a simple causal interpretation of these findings and reduces the possible confidence in such conclusions. There is a need for more research into the organisation and delivery of diabetes care services in general practice, preferably using patient level data; in order to better understand the impact of the different staffing configurations on patient outcomes.

## Background

In recent years there has been an increase in the contribution of non-medical health professions, nurses in particular, to the care of those with chronic illness in primary care in many countries. In this study we examine the association between nurse staffing and admissions for a range of chronic diseases in England, where nurse staffing levels in general practice have been linked to attainment of general practice performance targets for some chronic conditions[1].

Hospital admissions for complications of chronic conditions, such as asthma and diabetes, have been steadily increasing [2]. This represents a huge burden on healthcare systems. Delivering better quality primary care for patients with chronic conditions might lead to fewer hospital admissions. This has been confirmed in the USA, where better management in primary care was found to be associated with fewer emergency admissions for COPD [3]. Accordingly, non-elective admission rates can be a useful proxy measure (a valid indicator) of primary care quality.

One of the major goals of the UK National Health Service (NHS) in recent years has been to improve the quality of chronic disease management in primary care. In an effort to achieve this, a new national contract for general practitioners (GPs) was introduced in 2004 [4]. In addition to considering generic aspects of quality and organisational factors the new contract incentivised achieving quality care for a number of conditions which are assessed by the clinical performance targets identified and measured in the Quality and Outcomes Framework (QOF). The QOF records the practices performance on a number of dimensions of care process (e.g. proportion of diabetic patients whose blood pressure is checked) and intermediate outcomes (e.g. proportion of those with hypertension whose blood pressure falls below a target) for a range of chronic conditions (e.g. asthma, epilepsy, COPD, diabetes, hypertension, stroke).

Most of the work involved in delivering the performance targets has been delegated to nurses [5]. Nurses are also increasingly undertaking roles that have been the sole domain of doctors, such as prescribing. This role delegation has been particularly evident in the management of patients with stable chronic conditions that does not require extensive input from GPs and are the main disease areas incentivised in the new contract.

Evidence from observational studies has shown that better quality in UK primary care, as measured by the QOF scores attained, have been linked to a number of organisational factors. These included practice size, number of GPs and list size per full time equivalent (FTE) GP [6,7]. We previously found a significant association between registered nurses' (practice nurses/nurse practitioners) staffing levels and quality of care in a number of clinical areas that included COPD and diabetes [1]. Our aim in this study was to find out whether a similar association exists between nurse staffing levels and non-elective hospital admissions, as a quality measure external to QOF, for three clinical areas that are covered by the QOF: asthma, diabetes and COPD.

## Methods

### Ethical approval

Ethical approval was not required for this study.

### Sample

We used the same sample that was used previously when studying the relationship between nurse staffing and quality of care measured by QOF scores [1]. In 2005/2006 QOF data were collected from 8409 practices. A number of practices were excluded from this study because they were small (< 1000 patients), were lacking in condition specific registers, registers indicated no patients, there was at least a 50% mismatch between actual registers and the number of patients reported for individual indicators or where it was not possible to estimate the number of FTE practice nurses. This reduced the number of practices remaining in the analysis down to 7456.

### Data sources

Data were acquired from a number of sources. Data on admissions by practice were obtained from Dr. Foster Intelligence. This database contains records of all inpatient and day case care provided by the National Health Service (NHS) in England derived from the Commissioning Data Set. Admissions are coded according to the primary diagnoses associated to it, using the International Classification of Disease (ICD) version 10. We selected all non-elective admissions where the primary diagnoses was asthma, COPD or diabetes as classified by the Agency for Healthcare Research and Quality, Clinical Classification Software System for ICD10 [8]. We selected these three conditions because they represent QOF conditions where there are areas of significant activity by nurses in General Practice where the classifications to identify admissions were available. We extracted non-elective admissions data by each GP practice operating in England for the period April 2005 until end of March 2006 (the QOF reporting period). This allowed direct comparison with research on QOF outcomes for the same period [1].

Population and practice data were obtained from the Office of National Statistics and other sources were linked to each practice [6]. The number of FTE practice nurses employed by each practice was estimated using the method described by Griffiths and colleagues [1]. Data on the number of nurses employed by each practice was obtained from the healthcare specialist Binleys and the number of FTE practice nurses employed by PCTs from the NHS Workforce Projects Benchmarking database [9]. These data were used to calculate the FTE practice nurse estimate for each practice. A validation survey was conducted to check the accuracy of these estimates. There was good concordance between these estimates and FTE practice nurses actually employed by the practices sampled in the survey [1]. The estimate was sub-divided into quintiles ≤3038.01 patients per FTE practice nurse (n = 1421), 3038.02-3901.48 (n = 1421), 3901.49-4823.44 (n = 1422), 4823.45-6210.68 (n = 1420), ≥6210.69 (n = 1422) for the analysis purposes with a sixth category (n = 350) representing those practices that did not have a practice nurse.

### Analysis

A two-level multilevel model (practices nested within PCTs) was estimated using MLwiN; a widely used multilevel modelling software package [10]. A similar set of independent variables to those used in an earlier paper [1] was used. These included variables related to geographical area (population density and the Index of Multiple Deprivation [11], which considers a range of factors in the area where the practice is located: income, employment, health and disability, education, skills and training, barriers to housing and services, living environment, and crime) practice patients (% aged ≥ 65, % from a racial or ethnic minority), practice profile (list size per FTE GP, singled handed practice, Primary Medical Services Contract) and general practitioner profile (% ≥ 45 years of age, % female, % UK qualified). A bias adjustment variable was also used to identify practices where recorded register size differed from the number of patients contributing to QOF indicators [1].

We modelled three dependent variables: the number of patients experiencing one or more non elective admission, the number of patients with two or more admissions and the ratio of actual to expected number of people admitted. In the first two cases data are modelled at the practice level and adjustment for patient characteristics are based on aggregated data. Patient gender at the practice level was excluded from the models because it has high colinearity with other patient profile explanatory variables [1].

To adjust for patient characteristics at the individual level we used the indirect standardisation method [12] and modelled a Standardised Admission Ratio (SAR). For the SAR, patient level hospital episode data for gender, age (five year bands) and deprivation are used to estimate the expected number of non-elective admissions. Deprivation was estimated using the Carstairs index for the patient's area of residence, which is based on small local area census results considering factors such as low socioeconomic status, car ownership, overcrowding and unemployment [13]. In this model, risk adjustment for age, gender and deprivation takes place at the patient level and for this reason we did not adjust for age, gender and deprivation at the practice level. The SAR models were compared against the other model for similarities and differences to determine whether our conclusions were sensitive to the approach taken to risk adjustment.

The dependent variable for all models is a count (admissions) that has a Poisson distribution. A Poisson model with a log link function was therefore fitted to the data. To incorporate the fact that we were modelling the number of patients with one or more admissions, which is a function of the number of patients at risk, an offset was required [10]. In the case of all patients admitted, and patients with two or more admissions, the register size was used as the offset and the expected number of patients admitted was used in the third (SAR) model.

The register size is a product of practice prevalence (of the condition of interest) and the practice list size. Therefore neither prevalence nor practice list size were included in models using register size as the offset. Practice list size also has a very strong correlation with the expected admissions and for this reason was dropped from the set of independent variables fitted in the SAR model.

## Results

There were 56311 people admitted for asthma (mean 2289 per 100,000 on the practice register), 101782 for COPD (17692 per 100,000 on the register), and 33552 for diabetes (2015 per 100,000 on the register). The mean number per practice was 7.55 for asthma, 13.65 for COPD and 4.50 for diabetes. The mean number of patients per practice with two or more non-elective admissions was 0.81 for asthma, 3.05 for COPD and 0.68 for diabetes. Descriptive statistics for practice and patient characteristics are shown in Table 1.

**Table 1:** Practice and patient characteristics

Characteristic	Mean	Std. Deviation
Density (people per hectare 2001)	44.7	38.0
Index of Multiple Deprivation	25.9	17.0
% Patients ≥65 Years of age	15.1	5.1
% Patients who were members of a racial or ethnic minority	12.4	18.6
Size of practice population	6438	3897
List size per FTE GP	2183	928
% GPs ≥ 45 years of age	66.6	31.4
% Female GPs	32.3	28.3
% GPs qualified in UK	70.3	39.4
	**%**	**No**.
Single Handed Practice (yes)	23.6	1761
Primary Medical Services Contract (yes)	65.8	4906
	**Mean**	**Std. Deviation**
**Asthma**		
Estimated register size	376	250
Prevalence (%)	5.7	1.5
**Chronic Obstructive Pulmonary Disease**		
Estimated register sizeª	91	73
Prevalence (%)	1.4	0.8
**Diabetes**		
Estimated register size	230	143
Prevalence (%)	3.7	1.0

Note: N = 7456 unless indicated otherwise ^a ^n = 7446

Modelling the relationship between the non-elective admission rate and practice nurse staffing level revealed a mixed picture depending on the clinical condition.

For asthma, higher nurse staffing level was associated with lower admission rates. The association was significant overall (p < 0.001) and at all levels of nurse staffing (p < 0.001 or p < 0.01) compared to practices without a nurse, where the reduction in admissions increased up to a maximum for practice nurse staffing levels in the third quintile (list size 3902-4823 patients per FTE nurse) [table 2].

**Table 2:** Relationship between practice characteristics and number of patients experiencing one or more admissions (rate per no. of patients on the register)

	Asthma		COPD		Diabetes	
Characteristic	β	SE(β)	β	SE(β)	β	SE(β)
Intercept	-3.7800	0.0312	-1.8311	0.0270	-4.0069	0.0411
						
**Bias adjustment**						
						
Denominator used to estimate register	0.0084	0.0148	-0.0085	0.0078	-0.0222	0.0119
						
**Area**						
						
Density (people per hectare 2001)	0.0316^c^	0.0063	0.0309^c^	0.0050	0.0121	0.0081
Index of Multiple Deprivation	0.1125^c^	0.0061	0.0513^c^	0.0044	0.1082^c^	0.0075
						
**Patients**						
						
≥65 Yr of age	-0.0646^c^	0.0064	-0.0134^b^	0.0050	-0.1023^c^	0.0082
% member of racial or ethnic minority	0.0675^c^	0.0075	0.0089	0.0063	-0.0989^c^	0.0097
						
**Practice**						
						
List size per FTE GP	-0.0186^c^	0.0041	-0.0278^c^	0.0029	-0.0093^a^	0.0045
Single Handed Practice	0.0292	0.0171	0.0407^b^	0.0129	-0.0051	0.0215
Primary Medical Services Contract	-0.0128	0.0101	-0.0409^c^	0.0075	-0.0121	0.0127
						
**Family Practitioners**						
						
≥45 Yr of age	0.0066	0.0056	0.0008	0.0041	0.0014	0.0071
% Female GPs	-0.0096	0.0057	-0.0261^c^	0.0043	-0.0116	0.0072
% GPs qualified in UK	-0.0861^c^	0.0064	-0.0491^c^	0.0048	0.0105	0.0082
						
**Practice Nurse Staffing**						
						
List size per FTE practice Nurse(Quintiles)						
1st <3038.01	-0.1295^c^	0.0299	-0.0829^b^	0.0257	0.1269^b^	0.0413
2nd 3038.02-3901.48	-0.1313^c^	0.0296	-0.0600^a^	0.0255	0.1028^a^	0.0409
3rd 3901.49-4823.44	-0.1347^c^	0.0295	-0.0555^a^	0.0255	0.0790	0.0409
4th 4823.45-6210.68	-0.1091^c^	0.0294	-0.0410	0.0254	0.0962^a^	0.0408
5th 6210.69+	-0.0856^b^	0.0296	0.0138	0.0255	0.0991^a^	0.0409
No Practice nurse	0.0000		0.0000		0.0000	
χ^2 ^5df	35.13	p < .001	89.84	p < .001	12.64	p = .027
						
**Variance**						
						
PCT Level	0.0506	0.0048	0.0345	0.0032	0.0356	0.0038

df: degrees of freedom. ^a ^p < .05;^b ^p < .01; ^c ^p < .001

For COPD, higher practice nurse staffing level were similarly associated with lower non-elective admission rates (p < 0.001) [table 2]. There was a significant difference between all levels of practice nurse staffing above the 4^th ^quintile (4823-6210 list size per FTE nurse) and practices without a nurse (p < 0.01 or p < 0.05). The largest effect (β = -0.083) was for the highest level of nurse staffing (list size <3039 per FTE practice nurse). The effect size diminished in a linear fashion as nurse staffing levels fell, with no significant difference between practices without a nurse and those beyond the third quintile (list size 3902-4823 patients per FTE nurse).

For diabetes, however, higher levels of nurse staffing were significantly associated with an increased number of admissions (p < 0.05) [table 2]. Practices without a practice nurse performed better than those with a practice nurse for all levels of nurse staffing (p < 0.01 or p < 0.05) except the 3^rd ^quintile (3901-4823 list size per FTE nurse). The overall association with nurse staffing was noticeably weaker for diabetes (χ^2 ^= 12.63) than asthma (35.13) and COPD (89.84).

All continuous variables were standardised therefore we could calculate the increased risk associated with a change in the independent variable. For the nurse staffing variable the exponential of each β coefficient will give the altered risk of one of more admissions for each level of staffing (Quintiles) compared to practices that have no practice nurse.

For asthma, 14.4% more people experienced a non elective admission in practices without practice nurses compared to those practices in the third quintile (3901-4823 patients per practice nurse). If all practices were performing to the same level as those in the third quintile then the overall number of patients admitted would drop by 1.8%. For COPD there were 8.6% more patients admitted in practices without practice nurses compared to those practices in the first quintile (< 3038 patients per practice nurse) and if all practices were performing to that level the overall number of non-elective admissions would drop by 4.1%. For diabetes admissions, were 11.9% lower in practices without practice nurses compared to those practices in the first quintile (< 3038 patients per practice nurse). If all practices were performing to the same level as those without a practice nurse then the overall number of non-elective admissions would drop by 9.2%.

The model for patients with two or more admissions, was broadly similar, with lower levels of admissions associated with higher levels of nurse staffing for asthma (p < 0.05) and COPD (p < 0.001) [table 3]. For diabetes, the association between the rate of repeat admission and practice nurse staffing levels was not statistically significant (p = 0.062) [table 3].

**Table 3:** Relationship between practice characteristics and number of patients experiencing two or more admissions (rate per no. of patients on the register)

	Asthma		COPD		Diabetes	
Characteristic	β	SE(β)	β	SE(β)	β	SE(β)
Intercept	-5.0509	0.0552	-2.8036	0.0414	-5.1968	0.0733
						
**Bias adjustment**						
						
Denominator used to estimate register	-0.0485	0.0283	-0.0129	0.013	-0.056^b^	0.0211
						
**Area**						
						
Density (people per hectare 2001)	0.0378^c^	0.0114	0.0451^c^	0.0081	0.0249	0.1363
Index of Multiple Deprivation	0.1564^c^	0.0107	0.051^c^	0.007	0.1296^c^	0.0122
						
**Patients**						
						
% ≥65 Yr of age	-0.0594^c^	0.0116	-0.0142	0.008	-0.0702^c^	0.0139
% member of racial or ethnic minority	0.0656^c^	0.0132	0.0088	0.0099	-0.1081^c^	0.0157
						
**Practice**						
						
List size per FTE GP	-0.0159^a^	0.0073	-0.023^c^	0.0044	-0.0211^a^	0.0091
Single Handed Practice	-0.0158	0.0321	0.0319	0.0213	-0.0267	0.0386
Primary Medical Services Contract	-0.0081	0.0186	-0.0447^c^	0.0123	-0.0166	0.022
						
**Family Practitioners**						
						
% ≥45 Yr of age	0.0003	0.0104	0.0031	0.0068	0.0145	0.0125
% Female GPs	-0.0147	0.0106	-0.0166^a^	0.0071	-0.0136	0.0128
% GPs qualified in UK	-0.0897^c^	0.0119	-0.0534^c^	0.008	0.0254	0.0144
						
**Practice Nurse Staffing**						
						
List size per FTE practice Nurse(Quintiles)						
1st <3038.01	-0.1213^a^	0.0562	-0.1242^b^	0.0417	0.2064^b^	0.0751
2nd 3038.02-3901.48	-0.1220^a^	0.0556	-0.1079^b^	0.0413	0.1415	0.0748
3rd 3901.49-4823.44	-0.1104^a^	0.0555	-0.1013^a^	0.0413	0.1486^a^	0.0746
4th 4823.45-6210.68	-0.1054	0.0553	-0.0839^a^	0.0412	0.1377	0.0745
5th 6210.69+	-0.0533	0.0555	-0.038	0.0413	0.1248	0.0747
No Practice nurse	0		0		0	
χ^2 ^5df	13.2	p = .022	31.8	p < .001	10.51	p = .062
						
**Variance**						
						
PCT Level	0.062	0.0069	0.0322	0.0035	0.0338	0.0052

df: degrees of freedom. ^a ^p < .05;^b ^p < .01; ^c ^p < .001

The relationship between the SAR and practice nurse staffing for asthma and diabetes was similar to that obtained using the crude admission rate. Lower SARs for asthma were significantly associated with higher levels of practice nurse staffing (p < 0.05) [table 4]. For COPD, nurse staffing levels were not significantly associated with SARs [table 4].

**Table 4:** Relationship between practice characteristics and Standardised Admissions Ratio

Standardised Admission Ratio						
	Asthma		COPD		Diabetes	
Characteristic	β	SE(β)	β	SE(β)	β	SE(β)
Intercept	0.0324	0.0316	-0.0390	0.0279	-0.1188	0.0416
						
**Bias adjustment**						
						
Denominator used to estimate register	-0.0004	0.0148	0.0000	0.0079	-0.0027	0.0119
						
**Area**						
						
Density (people per hectare 2001)	0.0360^c^	0.0062	0.0307^c^	0.0050	0.0377^c^	0.0079
						
**Patients**						
						
% member of racial or ethnic minority	0.0660^c^	0.0072	-0.0386^c^	0.0063	-0.0011	0.0098
						
**Disease prevalence**						
						
Unadjusted prevalence	0.0849^c^	0.0054	0.1537^c^	0.0040	0.1041^c^	0.0070
						
**Practice**						
						
List size per FTE GP	-0.0018	0.0048	-0.0064	0.0034	0.0083	0.0053
Single Handed Practice	0.0011	0.0172	0.0106	0.0132	-0.0216	0.0220
Primary Medical Services Contract	0.0036	0.0100	0.0031	0.0076	0.0182	0.0128
						
**Family Practitioners**						
						
≥45 Yr of age	0.0005	0.0055	-0.0214^c^	0.0042	-0.0052	0.0071
% Female GPs	-0.0015	0.0056	-0.0010	0.0043	-0.0157^a^	0.0073
% GPs qualified in UK	-0.0681^c^	0.0064	-0.0490^c^	0.0049	-0.0369^c^	0.0082
						
**Practice Nurse Staffing**						
						
List size per FTE practice Nurse(Quintiles)						
1st <3038.01	-0.0695^a^	0.0294	0.0312	0.0255	0.1376^c^	0.0413
2nd 3038.02-3901.48	-0.0744^a^	0.0291	0.0272	0.0253	0.1085^b^	0.0410
3rd 3901.49-4823.44	-0.0927^b^	0.0290	0.0172	0.0253	0.0812^a^	0.0409
4th 4823.45-6210.68	-0.0733^a^	0.0289	0.0271	0.0252	0.0948^a^	0.0409
5th 6210.69+	-0.0690^a^	0.0291	0.0458	0.0253	0.0929^a^	0.0410
No Practice nurse	0.0000		0.0000		0.0000	
						
χ^2 ^5df	11.41	p = .044	10.01	p = .075	15.65	p < .001
						
**Variance**						
						
PCT Level	0.0655	0.0060	0.0516	0.0046	0.0452	0.0046
						

df: degrees of freedom. ^a ^p < .05;^b ^p < .01; ^c ^p < .001

Across all three models there were consistent associations between admissions and the Index of Multiple Deprivation (positive), Density (positive) and list size per FTE GP (negative) [table 2, 3, 4]. The relationship between percentage of patients from a racial or ethnic minority on the practice list and admissions varied depending on the clinical condition and model. Practices with more GPs qualified in the UK had fewer admissions. Single handed practices and type of contract were only significant for COPD. Single handed practices had significantly higher rates of admissions as did practices which had not signed up to the primary medical services contract.

Because the relationships observed between nurse staffing and admissions might be a product of clinical team size relative to workload, as opposed to nurse staffing per se, we calculated the list size per member of the clinical team (GPs and nurses) and the ratio of practice nurses to GPs [table 5]. There was no significant association between list size per clinical team member and admissions but significant associations between the nurse to GP ratio and admissions which were similar to those observed for list size per FTE practice nurse. The more nurses per GP for asthma and COPD the fewer admissions compared to those practices with no nurses while practices with no nurses had fewer admissions for diabetes compared to those with nurses although there was no clear trend for increasing admissions as the nurse to doctor ratio increased for those practices with nurses.

**Table 5:** Relationship between nurse to GP ratios and number of patients experiencing one or more admissions (rate per no. of patients on the register)

	Asthma		COPD		Diabetes	
Characteristic	β	SE(β)	β	SE(β)	β	SE(β)
List size per FTE GP and PN	0.0090	0.0056	0.0057	0.0043	-0.0056	0.0073
						
*PN to GP Ratio*						
No Practice nurse	0.0000		0.0000		0.0000	
1st .09 to .32	-0.0831^b^	0.0312	-0.0066	0.0264	0.0880^a^	0.0426
2nd .32 to .42	-0.0995^b^	0.0311	-0.0300	0.0265	0.0829	0.0426
3rd .42 to .53	-0.0994^b^	0.0313	-0.0437	0.0265	0.0445	0.0429
4th .53 to .70	-0.1062^c^	0.0311	-0.0811^b^	0.0265	0.0995^a^	0.0427
5th .70 and over	-0.1340^c^	0.0314	-0.0624^a^	0.0266	0.1002^a^	0.0429
						
χ^2 ^5df	22.12	p < .001	52.07	p < .001	16.53	p = .005

df: degrees of freedom. ^a ^p < .05;^b ^p < .01; ^c ^p < .001 (other model parameters estimated as per table 2, not reported)

## Discussion

### Main findings

We found evidence of an association between nurse staffing levels in English general practices and non-elective hospital admissions for asthma, COPD and diabetes. The relationships seem to be associated with the skill mix (ratio of nurses to doctors) rather than the size of the clinical workforce relative to the number of patients.

Patients registered with practices employing more nurses were less likely to have a non-elective admission related to their asthma and COPD. For COPD, the significance of the association varied from one model to another. Where significant, (2/3 models) the association was negative with higher nurse staffing level associated with lower admissions for COPD. For diabetes, however, the association was positive and showed significance in two of the three models fitted. This suggests that although higher nurse staffing levels have been shown to be associated with better compliance with processes of care and better intermediate clinical outcomes resulting in achieving higher QOF scores [1], its association with non-elective hospital admissions is likely to be dependent on other factors including the specific disease, service configurations and patient related factors not included in our models.

Contradictory evidence exists in the literature regarding whether the attainment of higher QOF scores is associated with positive effect on morbidity, mortality, hospital referrals and non-elective admissions [14-17]. Although the QOF might accurately assess the process of care, it is questionable whether the same is possible for clinical outcomes, despite some intermediate outcomes being included in the framework. This is in part due to the lack of adjustment for case mix when QOF rewards are calculated. Evidence from Canada also suggests that the relationship between performance and the actual health gains is questionable [18]. There is also controversy around the appropriateness and evidence-base of the targets relating to the control of acetylated haemoglobin (HbA1c) levels in diabetic patients that needs to be achieved in general practice [19]. We previously found that higher nurse staffing levels were associated with attainment of higher QOF scores in four out of eight clinical domains and with performance on specific indicators of intermediate clinical outcomes within the QOF clinical domains [1]. Using hospital non-elective admissions as an indicator of the quality of care delivery in general practice offers an external measure that can be useful in validating QOF performance measures. It can also be taken as a proxy measure of clinical outcomes.

We found partial support for the relationship between staffing and quality that we observed previously when using this external measure. In this study, we found that, for asthma, intermediate nurse staffing levels in the 2^nd ^and 3^rd ^quintiles (3038-3901 and 3901-4824 patients per FTE practice nurse) had the lowest admission rates in the crude and the SAR models respectively. This might indicate that an optimal mix of GPs and nurses, that does not require the highest levels of nurse staffing, could be the best strategy for delivering care to asthma patients in general practice.

However, simple changes in staff-mix are not sufficient without consideration of the context in which people work and the organisational factors related to that [20]. Hence, the variation in the relationship between higher nurse staffing levels and admission rates across the three clinical areas may relate to variations in the activity of nurses and/or how services are locally organised in those areas. This further confirms our earlier conclusion that there is a need to investigate the configuration of services and deployment of nurses more specifically [1].

While higher levels of nurse staffing were associated with better QOF performance for diabetes, including levels of HbA_1C, _in our previous study[1] they were associated with higher levels of admissions here. Although we did not directly assess the association between QOF performance and admissions in this study, this finding does confirm that the relationship is not a straightforward one. There are known associations between several socioeconomic and patient related factors and developing diabetes and its complications that are independent of the access to and quality of care provided. The National Diabetes Audit revealed that "the most deprived in the UK are two and a half times more likely to have diabetes" and that "complications of diabetes such as heart disease, stroke and kidney damage are three and a half times higher in the lower socio-economic groups" [21]. Reid and colleagues also found that patient factors were the strongest predictor of the large variation in all admission rates between 120 London general practices, especially for emergency admissions [22]. We have attempted to control for the socioeconomic factors, including deprivation at the practice level, as well as patient related factors, in the SAR. However, there are other patient related factors, like complexity of the condition and patient adherence to medication that can contribute to the likelihood of a diabetes related admission, which we were unable to include in the model.

### Strengths and limitations

This study includes data covering the vast majority of patients in English general practice. Although we excluded some practices, our study has examined evidence from the vast majority of English general practices providing care to 48 million patients. We have controlled for potential confounding variables but observational studies such as this cannot account for unmeasured factors. It may be that higher nurse staffing is associated with other unmeasured attributes of quality within the practice and if this is the case increasing nurse staffing will not bring benefits unless these factors are attended to. What remains unclear is whether there is a causal relationship at all.

We have used the rate of hospital non-elective admissions as an indicator of quality of primary care delivered to patients; however although rates of hospital non-elective admission for some chronic conditions are possible indicators of the quality of care, they should be interpreted in conjunction with measures of population composition and deprivation. Hospital non-elective admission rates are likely to be confounded by socioeconomic characteristics of the population, case-mix and secondary care characteristics (e.g. hospital policies) which are not under the control of the primary care providers. Lack of adjustment for this confounding can result in primary care trusts and general practitioners being penalised for patterns of service use that they cannot directly control [2]. This was echoed by Giuffrida et. al. [23] who argued that using admission rates for asthma, diabetes and epilepsy as an indicator of primary care quality can be misleading. In our analysis, we have not controlled for secondary care related factors that might impact on admissions.

The approaches used to model the data have their own advantages and disadvantages. The SAR model allows for some adjustment at the patient level albeit using a simple model whereas the admissions models are based on data collected solely on practices therefore only suffers from model misspecification at that level. There could be multiple reasons why some practices have more single admissions than others that go beyond the care they provide and there may be a stronger case for using subsequent rather than first admissions as a proxy measure of quality [14]. In our findings whereas the relationship between nurse staffing and diabetes non-elective admissions was significantly positive in the models for one or more admissions, the overall association was not statistically significant in the repeat admissions model although in all cases the relationships were broadly similar across all models. The lack of consistency in relationships seems to indicate that nonetheless there is residual confounding.

We did not explore the cost implications of the variations in staffing observed. The absolute numbers of admissions across these conditions is high and so the relatively small reductions in rates of admissions translate into large absolute numbers of people. However when considering the numbers of people admitted per practice it seems clear that, even if causality was assumed, employing nurses to reduce admissions may be an expensive solution and could only be justified if there was a wide benefit in quality associated with a sufficiently large group of patients or across several conditions. However, hospital admissions are expensive and so economic gains through prevented admissions could also be large.

## Conclusions

The association between practice nurse staffing levels and non-elective admission rates was variable across the three clinical areas studied, namely asthma, COPD and diabetes. This variation may relate to variations in activity or effectiveness of nurses in those areas and future research needs to investigate the configuration of services and deployment of nurses more specifically. While the findings for asthma and COPD admissions lend some support to those of controlled trials of nurse for doctor substitution, further research is required to determine if the relationship is causal. The relationship between nurse staffing and diabetes non-elective admissions was less clear but tended toward an unfavourable one (i.e. more nurses more admissions). This may be confounded by variation in service configurations such as shared care between hospital and general practice. Broadly it may be that admissions for asthma and COPD are sensitive to care delivered in general practice whereas currently admissions for diabetes uncontrolled patient and service factors dominate, masking any benefits arising from quality in primary care. Different service configuration models need to be examined and their impact on patient outcomes need to be evaluated to identify the best strategy for using/deploying the available human resources for each clinical area as there is no "one (list) size (per practice nurse) fits all" solution. Optimal levels of nurse staffing need to be assessed according to local needs and service design.

The results of this study show that there is an association between the level of practice nurse staffing and the rates of hospital non-elective admissions for asthma, diabetes and COPD but the relationship is inconsistent. For asthma, and COPD, higher nurse staffing levels were associated with lower rates of hospital admissions. This may have implications for human resource planning in general practice, where there might be a case for investing in more nurses to deliver care for patients with these conditions. For diabetes, however, the link was generally unfavourable, despite earlier evidence associating nurse staffing with better care quality. The inconsistent relationship between nurse staffing and patient outcomes across the different conditions and the fact that for diabetes the relationship between staffing and outcomes was in a different direction from the association between staffing and care quality, highlights the need to avoid making a simple causal interpretation of these findings and reduces the possible confidence in such conclusions. There is a need for more research into the organisation and delivery of diabetes care services in general practice, preferably using patient level data; in order to better understand the impact of the different staffing configurations on patient outcomes.

## Competing interests

The authors declare that they have no competing interests.

## Authors' contributions

PG, TM and SJ designed the study. TM undertook the data linkage and managed and analysed the data. DD, TM, SJ and PG interpreted the results. DD and TM wrote the first draft of the paper and PG undertook later revisions. All authors contributed to the final draft.

## Pre-publication history

The pre-publication history for this paper can be accessed here:

http://www.biomedcentral.com/1472-6963/10/276/prepub
